# Simulated CO_2_-induced ocean acidification for ocean in the East China: historical conditions since preindustrial time and future scenarios

**DOI:** 10.1038/s41598-019-54861-0

**Published:** 2019-12-06

**Authors:** Han Zhang, Kuo Wang

**Affiliations:** 1 Zhejiang Climate Center, Hangzhou, Zhejiang 310017 China; 2 Department of Atmospheric Sciences, School of Earth Sciences, Zhejiang University, Hangzhou, Zhejiang 310027 China

**Keywords:** Carbon cycle, Climate and Earth system modelling

## Abstract

Since preindustrial times, as atmospheric CO_2_ concentration increases, the ocean continuously absorbs anthropogenic CO_2_, reducing seawater pH and $[{{\rm{C}}{\rm{O}}}_{3}^{2-}]$, which is termed ocean acidification. We perform Earth system model simulations to assess CO_2_-induced acidification for ocean in the East China, one of the most vulnerable areas to ocean acidification. By year 2017, ocean surface pH in the East China drops from the preindustrial level of 8.20 to 8.06, corresponding to a 35% rise in [H^+^], and reduction rate of pH becomes faster in the last two decades. Changes in surface seawater acidity largely result from CO_2_-induced changes in surface dissolved inorganic carbon (DIC), alkalinity (ALK), salinity and temperature, among which DIC plays the most important role. By year 2300, simulated reduction in sea surface $[{{\rm{C}}{\rm{O}}}_{3}^{2-}]$ is 13% under RCP2.6, contrasted to 72% under RCP8.5. Furthermore, simulated results show that CO_2_-induced warming acts to mitigate reductions in $[{{\rm{C}}{\rm{O}}}_{3}^{2-}]$, but the individual effect of oceanic CO_2_ uptake is much greater than the effect of CO_2_-induced warming on ocean acidification. Our study quantifies ocean acidification induced by anthropogenic CO_2_, and indicates the potentially important role of accelerated CO_2_ emissions in projections of future changes in biogeochemistry and ecosystem of ocean in the East China.

## Introduction

Atmospheric CO_2_ concentration has reached 407.44 ± 0.10 ppm (parts per million) by year 2018, increased by 46% since preindustrial time^1^, which is mainly due to human activities of fossil fuel burning and land use changes. Observational-based estimates show that, during year 1750 and 2017, total anthropogenic CO_2_ emission is 660 ± 95 PgC (1 PgC = 10^15^ grams of carbon = 1 billion tons of carbon)^2^. About 42% of these emissions stayed in the atmosphere, meanwhile, about 25% and 33% of the emissions were absorbed by the ocean and terrestrial biosphere, respectively^2^.

The rise of atmospheric CO_2_ concentration results in global warming through trapping long wave radiation, a process known as greenhouse effect^3–6^. Global warming could alter physical, chemical, and biological processes in the ocean^7–10^. Oceanic uptake of CO_2_ could buffer part of the global warming; however, not only global warming, the penetration of anthropogenic CO_2_ would also perturb ocean chemistry by making seawater more acidic, which is termed as ocean acidification^11^.

Generally, ocean acidification is caused primarily by oceanic CO_2_ uptake from the atmosphere. In addition, especially in the coastal seas, there are some other factors that could also lead to the acidification of seawater. For example, increasing input of anthropogenic nitrogen to the ocean, and the resultant changes in organic matter production, oxidation, and deoxygenation, may have effects on ocean acidification^12^. However, many recent studies suggested that impacts of biological nitrogen assimilation and release on ocean acidification are negligible compared with impacts of CO_2_ absorption^13,14^. Variations in riverine carbon fluxes to the ocean could either accelerate or offset seawater acidity in coastal areas, revealing large uncertainties^15^. Also, some processes in the ocean CaCO_3_ cycle, including calcification and ballast effects, could also trigger feedbacks to ocean acidification^16–18^. This study will focus on the individual effect of oceanic CO_2_ uptake on ocean acidification, which is generally considered a key process affecting seawater acidity and the ocean carbon cycle in the East China. In addition, we also assess the effect of CO_2_-induced warming on ocean acidification, and compare the strength of individual effects of oceanic CO_2_ uptake and CO_2_-induced warming on ocean acidification in the East China.

Seawater pH is known as a measurement to quantify the degree of ocean acidification. Since the industrial revolution, sea surface pH (pH = −log_10_[H^+^]) has dropped by about 0.1 units by year 2013, corresponding to an increase of 26% in hydrogen ion concentration ([H^+^])^19^. The current pH reduction rate is likely to be the highest during the past hundreds of thousands of years^20^. The elevated hydrogen ion concentration tended to reduce carbonate ion concentration ($[{{\rm{C}}{\rm{O}}}_{3}^{2-}]$) via:

1 $${{\rm{H}}}^{+}+{{\rm{CO}}}_{3}^{2-}\to {{\rm{HCO}}}_{3}^{-}$$

The reduction in carbonate ion concentration would lower seawater calcium carbonate (CaCO_3_) saturation state (Ω) for aragonite or calcite (two different polymorphs of CaCO_3_), which is defined as:

2 $$\Omega ={[{\rm{Ca}}}^{2+}{][{\rm{CO}}}_{3}^{2-}]/{{\rm{K}}}_{{\rm{sp}}}^{\ast }$$

where ${{\rm{K}}}_{{\rm{sp}}}^{\ast }$ is the stoichiometric solubility product constant with respect to aragonite or calcite^3,21^.

The main concern of ocean acidification originates from the potentially adverse impacts of CaCO_3_ saturation state reductions on marine calcifying organisms, which use CaCO_3_ to form their skeletons or shells. For instance, reduced Ω could decrease calcification rate of calcifying organisms^22–28^, and increase CaCO_3_ dissolution rate^29–34^, making their skeletons or shells vulnerable. In addition, by conducting meta-analyses of case studies, Jin *et al*. (2015) suggested, during the past decade, the echinoderm/microbenthic productivity has been altered in the seas around China, which has implications for the effects of ocean acidification on marine ecosystem^35^. Concluded from laboratory experiments, Liu and He (2012) also provided evidences of the potentially important impacts of ocean acidification on metabolic processes of some calcifying organisms in the China seas^36^. Calcifying organisms in the China seas could be of major ecological and economic importance. China is known as one of the most important countries of marine aquaculture industry. At year 2016, aquaculture production (excluding aquatic plants) from China is 4.97 × 10^7^ t, accounting for about 61% of the global total aquaculture production, and large quantities of the marine aquaculture production are calcifying organisms^37^. Therefore, acidification in the China seas could lead to reductions in the aquaculture production and the consequent economic losses, meanwhile, posing threats to marine ecosystems.

For lack of historical observational data and the requirement for future projections in ocean chemistry fields, numerical models were used to investigate the changes in ocean acidification and biogeochemical processes in previous studies. For example, modeling studies show consistently that the drop in sea surface mean pH since preindustrial times is about 0.1^11,38,39^, which is compared well with observational-based estimates^19^. By forcing the Lawrence Livermore National Laboratory ocean general-circulation model under IPCC SRES scenario, Caldeira and Wickett (2005) indicated that ocean surface pH could be reduced by 0.3–0.5 units by the end of this century^40^. By forcing 13 ocean-carbon cycle models under IPCC IS92a scenario, Orr *et al*. (2005) projected that surface seawater would be undersaturated with respect to aragonite in Mid-21st century^38^.

In addition to the global scale, projections of future ocean acidification for specific spots of ocean in the East China are also provided in some other studies^41–44^. For example, Chou *et al*. reported that under the IPCC IS92a emission scenario, under the total effects of eutrophication and elevated atmospheric CO_2_, the bottom water of the Yangtze River plume area would be undersaturated with respect to aragonite (Ω_A_≈0.8) by the end of this century, threatening the benthic ecosystem^43^. Zhai, using a predicted scenario that atmospheric CO_2_ increases by 100 ppm for the 2050 s since present, proposed that half of the Yellow Sea benthos would be covered by acidified seawater having a critical Ω_A_ of less than 1.5^41^. In addition, under the IS92a scenario, Xu *et al*. proposed that by year 2100, surface seawater with Ω_A_ > 2.0 would disappear over most of the ocean area in BoHai and Yellow seas of China^42^.

Compared to other models that previous studies used, UVic ESCM (the University of Victoria Earth System Climate Model) as a coupled climate-carbon cycle model of intermediate complexity, could simulate climate and ocean chemistry fields in millennia timescales with lower computational expenses. Meanwhile, the UVic model could reasonably capture observed key variables in global climate^45^, the ocean carbon cycle^46–48^, and historical oceanic uptake of carbon and its isotopes^49^. Refer to Methods section for detailed descriptions of the UVic model.

In this study, we extend previous studies by using UVic model to quantify ocean acidification induced by anthropogenic CO_2_ for ocean in the East China (115–130°E, 20–40°N). Usually, previous studies would only focus on analysing the changes in seawater acidity, e.g., seawater pH; this study further quantifies the impacts of changes in ocean chemistry (i.e., DIC, ALK, salinity and temperature) on ocean acidification, as well as analyses the spatial heterogeneity of ocean acidification. In addition, we investigate the effects of CO_2_-induced warming on different ocean chemistry fields, and compare the strength of individual impacts of oceanic CO_2_ uptake and CO_2_-induced warming on ocean acidification, which was ignored by previous relevant studies. Furthermore, in this study, we analyse the nonlinearity relationship between atmospheric CO_2_ scenario used and ocean acidification, which enable us to have a better estimate of the extent of ocean acidification in the East China under different CO_2_ emission policies. We aim to further our understanding of the role played by accelerated anthropogenic CO_2_ emissions in the carbon cycle of ocean in the East China, which is also important for reliable projections of future changes in marine biogeochemistry and ecosystem in west Pacific.

## Results

**Figure 1:**
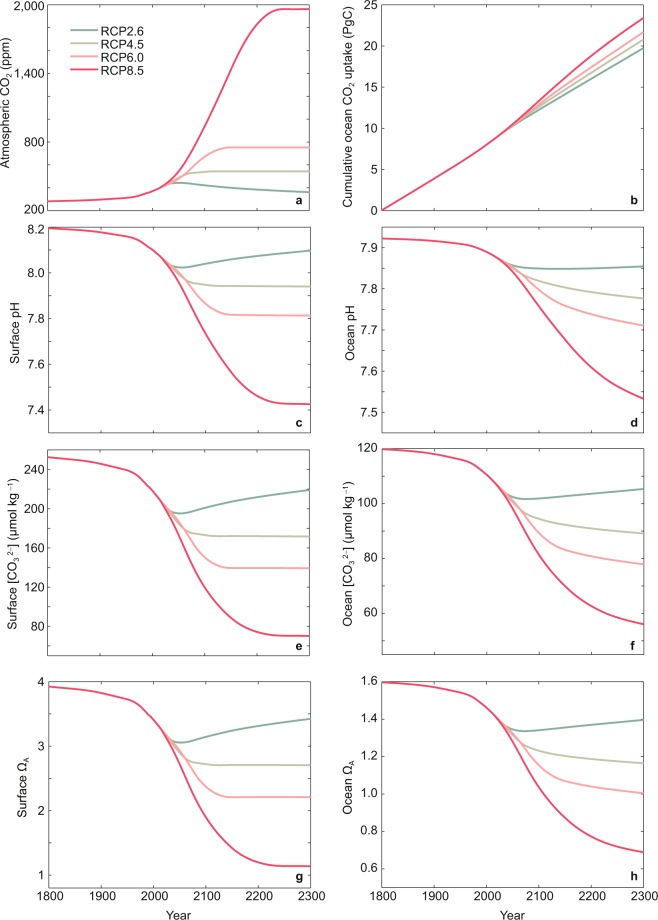
(**a**) Prescribed atmospheric CO_2_ concentration and model-simulated time series of annual mean variable of (**b**) cumulative ocean CO_2_ uptake, (**c**) ocean surface pH, (**d**) ocean mean pH, (**e**) ocean surface $[{{\rm{C}}{\rm{O}}}_{3}^{2-}]$, (**f**) ocean mean $[{{\rm{C}}{\rm{O}}}_{3}^{2-}]$, (**g**) ocean surface Ω_A_, (**h**) ocean mean Ω_A_ in the East China. Results are shown for the four simulations using the four RCP scenarios depicted in Methods section.

To quantify the effect of increasing atmospheric CO_2_ concentration on ocean acidification in the East China, a series of 500-year Earth system model simulations are designed. From year 1800 to 2017, all model simulations are forced by observational-based atmospheric CO_2_ concentration. After 2017, simulations are forced by Representation Concentration Pathway scenarios (RCPs, including RCP2.6, RCP4.5, RCP6.0, and RCP8.5) and their extensions up to year 2300 (Fig. 1a). To quantify the influences of CO_2_-induced warming on ocean acidification in the East China, we conducted an additional set of simulations in which CO_2_-induced warming is not allowed to affect the ocean carbon cycle. Refer to Methods section for detailed descriptions of the model and simulation experiments.

In the following, we first present results from the simulations including CO_2_-induced warming effects on the ocean carbon cycle. Then, in the “Impacts of CO_2_-induced warming on ocean acidification in the East China” section, by comparing the results from simulations with and without CO_2_-induced warming, we analyse the effects of CO_2_-induced warming on ocean acidification in the East China.

### Model evaluation

UVic-simulated global oceanic CO_2_ uptake during the historical period since preindustrial time is consistent with observational-based estimates reported by IPCC AR5^8^ (Table 1). For example, simulated cumulative oceanic CO_2_ uptake during preindustrial time-year 2011 is 147 PgC, within the observational range of 155 ± 30 PgC reported by IPCC AR5^8^ (Table 1). Model-simulated averaged oceanic CO_2_ uptake during 2002–2011 is 2.4 PgC yr^−1^, which compares well with the observed value of 2.4 ± 0.7 PgC yr^−1^ (Table 1). Model-simulated atmospheric CO_2_ concentration is also consistent with observations. For example, simulated annual mean atmospheric CO_2_ concentration at year 2017 is 405.0 ppm (Fig. 1a), compared well with observational-based estimate of 405.0 ± 0.1 ppm^1^.

**Table 1:** Model-simulated global oceanic CO_2_ uptake compared with observational-based estimates reported in IPCC AR5, which show uncertainties as 90% confidence intervals^8^.

	UVic ESCM	IPCC AR5
Preindustrial-2011 cumulative (PgC)	147	155 ± 30
1980–1989 average (PgC yr^−1^)	1.8	2.0 ± 0.7
1990–1999 average (PgC yr^−1^)	2.0	2.2 ± 0.7
2000–2009 average (PgC yr^−1^)	2.3	2.3 ± 0.7
2002–2011 average (PgC yr^−1^)	2.4	2.4 ± 0.7

Shown in the table are the accumulated oceanic CO_2_ uptake during preindustrial time-year 2011, and averaged oceanic CO_2_ uptake during the 1980s, 1990s, 2000s, and the decade since 2002.

Model-simulated carbon-related tracers are also compared with observed estimates from the Global Ocean Data Analysis Project (GLODAP)^50^. As shown in Supplementary Fig. S1, simulated vertical profiles of dissolved inorganic carbon (DIC) and alkalinity (ALK) for ocean in the East China agree well with observational-based estimates. In addition, the UVic model can capture the observed large-scale distributions of key tracers for the global ocean, as well as different ocean basins (refer to Supplementary Material of Cao *et al*.^48^).

### Ocean acidification in the East China: historical conditions since preindustrial time

Under the CO_2_ concentration scenario depicted in Fig. 1a and Supplementary Fig. S2a, by year 2017, the atmospheric CO_2_ concentration increases to 404 ppm (see Supplementary Table S1). With the increasement in atmospheric CO_2_ concentration, the ocean continuously absorbs anthropogenic CO_2_ from the atmosphere, leading to acidification in the global ocean (see Supplementary Fig. S3). As shown in Fig. S3, generally, the mid-latitude ocean surface shows greater decreases in pH, $[{{\rm{C}}{\rm{O}}}_{3}^{2-}]$ and Ω_A_ than the tropical and high latitude ocean surface.

As part of the mid-latitude ocean, ocean surface in the East China suffers greater acidification relative to sea surface in low and high latitudes from preindustrial time to year 2017. For example, by year 2017, sea surface mean pH in the high and low latitudes both dropped by 0.11 units, while sea surface pH in the East China dropped by 0.13 units, corresponding to a 35% rise in [H^+^] (Fig. 1c, Supplementary Figs. S2b, S3a and Table S1). Over the same time period, sea surface mean $[{\rm{C}}{{\rm{O}}}_{3}^{2-}]$ in the low latitudes reduced by 16%, contrasted to reductions by 18% for ocean in the East China (Fig. 1e, Supplementary Fig. S3b, and Table S1). This study will focus on analysing ocean acidification conditions in the East China (unless otherwise stated), in which ecosystems could be especially vulnerable to ocean acidification, triggering important effects on global fishery and marine aquaculture industries.

**Figure 2:**
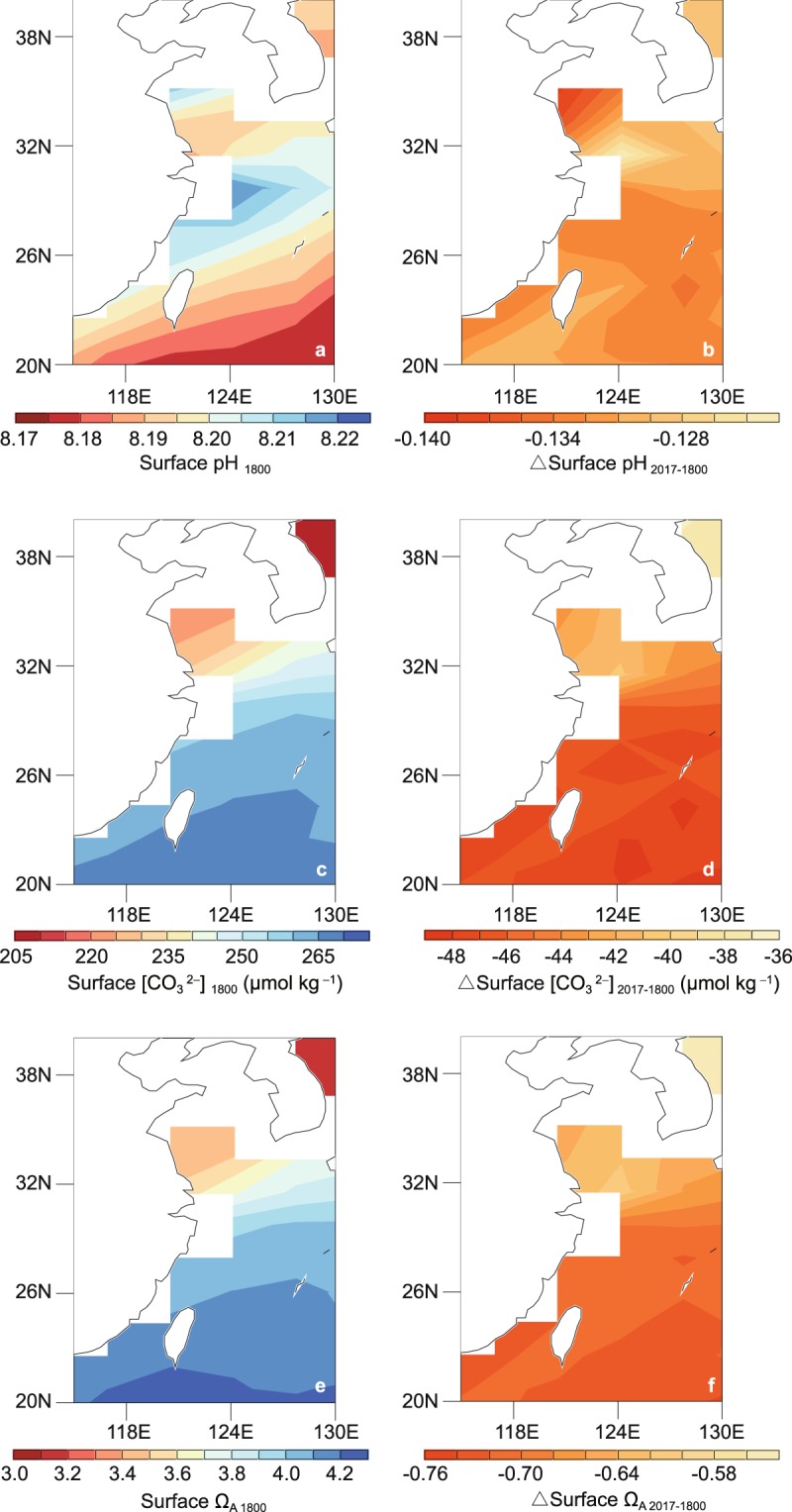
Spatial distributions of simulated ocean surface (**a,b**) pH, (**c**,**d**) $[{{\rm{C}}{\rm{O}}}_{3}^{2-}]$, and (**e**,**f**) Ω_A_ in the East China. Results shown in (**a**,**c**,**e**) are for year 1800 and (**b**,**d**,**f**) are for the changes at year 2017 relative to 1800. The figures are generated using UV-CDAT version 2.5.0 (http://uvcdat.llnl.gov/).

**Figure 3:**
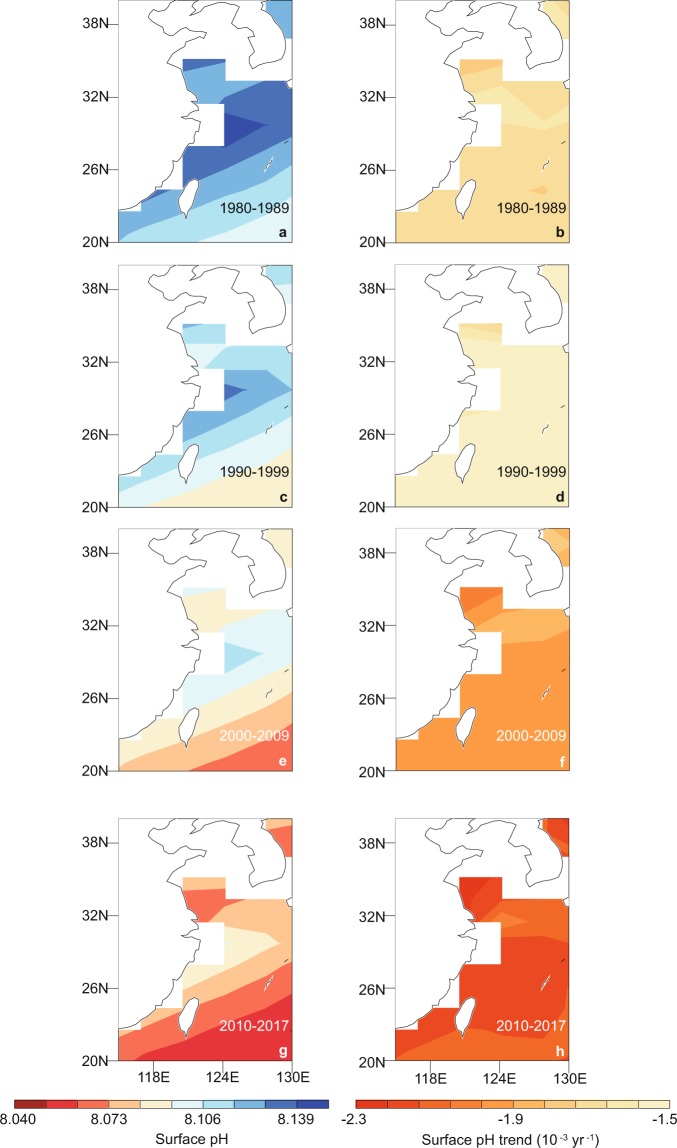
Spatial distributions of simulated (**a**,**c**,**e**,**g**) decadal mean and (**b**,**d**,**f**,**h**) decadal mean trend of ocean surface pH in the East China. Results are shown for years (**a**,**b**) 1980–1989, (**c**,**d**) 1990–1999, (**e**,**f**) 2000–2009, (**g**,**h**) 2010–2017, respectively. The figures are generated using UV-CDAT version 2.5.0 (http://uvcdat.llnl.gov/).

Figs. 2 and 3 show the simulated spatial distributions of the trend in ocean acidification in the East China from 1800 to 2017. Surface pH in the Yellow Sea shows a greater decrease relative to rest of the ocean in the East China, where surface $[{\rm{C}}{{\rm{O}}}_{3}^{2-}]$ and Ω_A_ reduce less. The different spatial distributions of the trends in ocean surface pH, $[{\rm{C}}{{\rm{O}}}_{3}^{2-}]$ and Ω_A_ are mainly due to different thermodynamic dependence of pH and $[{{\rm{C}}{\rm{O}}}_{3}^{2-}]$ on temperature^32^. With the growth in atmospheric CO_2_ content, the ocean’s continuous absorption of CO_2_ leads to the exacerbation of ocean acidification with faster speed (Fig. 3). For instance, simulated results show the reductions in sea surface pH are faster in the last 20 years (years 2000–2009 and 2010–2017) relative to years 1980–1999 (Fig. 3f,h, Supplementary Fig. S2b).

**Figure 4:**
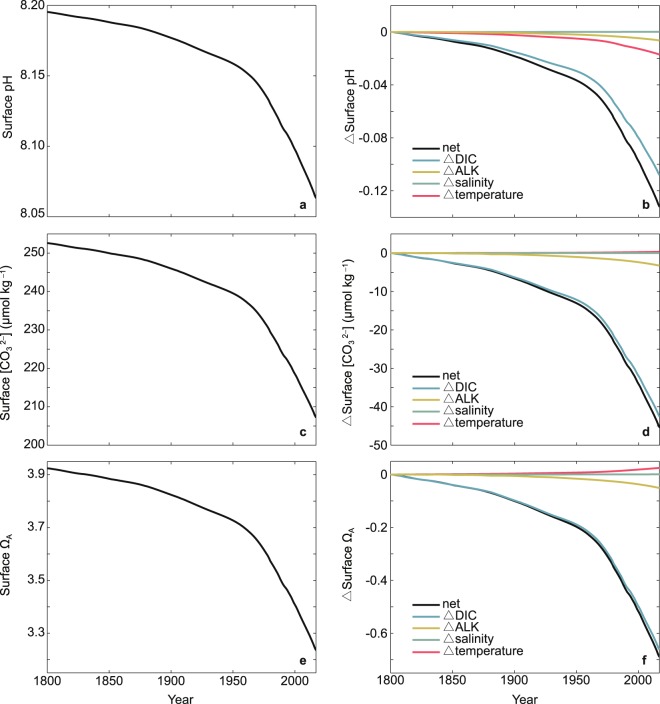
Time series of simulated ocean surface (**a**,**b**) pH, (**c**,**d**) $[{{\rm{C}}{\rm{O}}}_{3}^{2-}]$, and (**e**,**f**) Ω_A_ in the East China from year 1800 to 2017. Results shown in (**a**,**c**,**e**) are for annual mean, and (**b**,**d**,**f**) are for the individual effects of changes in dissolved inorganic carbon (DIC), alkalinity (ALK), salinity and temperature on the corresponding fields.

**Figure 5:**
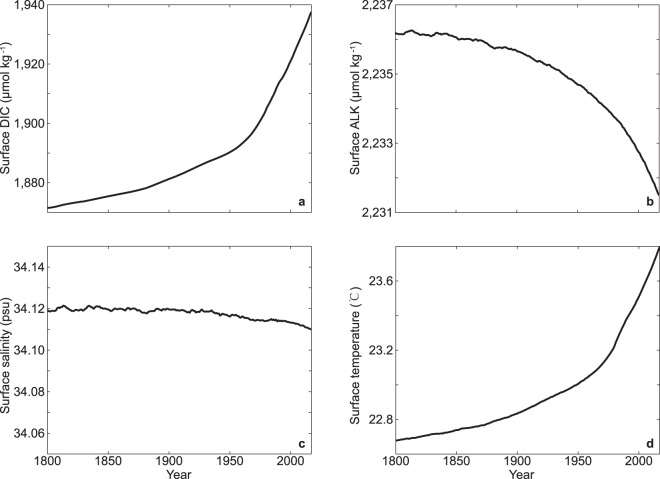
Time series of simulated annual mean ocean surface (**a**) dissolved inorganic carbon (DIC), (**b**) alkalinity (ALK), (**c**) salinity, (**d**) temperature in the East China from year 1800 to 2017.

Changes in seawater acidity in the East China mainly result from CO_2_-induced changes in ocean DIC, ALK, salinity and temperature (Fig. 4, refer to Methods section for detailed descriptions of the analysis of ocean chemistry fields). As shown in Figs. 4 and 5, the decreases of sea surface pH, $[{{\rm{C}}{\rm{O}}}_{3}^{2-}]$ and Ω_A_ are largely a result of rising surface DIC, driven by the continuous oceanic CO_2_ uptake. For example, by year 2017, the increase of sea surface DIC accounting for 82%, 94%, and 96% of the reductions of surface pH, $[{{\rm{C}}{\rm{O}}}_{3}^{2-}]$ and Ω_A_, respectively (Fig. 4). The rising temperature due to global warming have different influences on sea surface pH and Ω_A_, mainly as a result of different thermodynamic dependences of these two variables on temperature. For instance, by year 2017, the rising temperature accounting for 13% and −4% of the decreases in sea surface pH and Ω_A_, respectively (Figs. 4 and 5). The reduction in sea surface alkalinity is mainly due to changes in ocean CaCO_3_ cycles, adding to the reductions in surface pH, $[{{\rm{C}}{\rm{O}}}_{3}^{2-}]$ and Ω_A_ (Figs. 4 and 5). Changes in sea surface salinity are relatively small and the effects of surface salinity changes on ocean acidification are nearly negligible (Figs. 4 and 5).

Different spatial distributions of changes in sea surface DIC, ALK, salinity and temperature could have implications for the distributions of changes in surface pH, $[{{\rm{C}}{\rm{O}}}_{3}^{2-}]$ and Ω_A_^17,48^. For example, surface pH in the Yellow Sea shows a greater decrease (1800–2017) relative to the rest of ocean in the East China, which is largely due to the rapid elevation in surface temperature, while surface DIC and ALK over the Yellow Sea does not show relatively significant trends (Fig. 2b, Supplementary Fig. S4).

In previous projects and studies, observational-based estimates of ocean acidification for specific areas in the East China have also been reported. For instance, field surveys conducted by Zhai at years 2012, 2015 and 2016, showing acidified seawaters with critical Ω_A_ of less than 1.5 in the Yellow Sea^41^. Whereas in our simulated results, ocean mean Ω_A_ (2012–2016 average) in the East China is about 1.4, consistent with the data-based estimates from Zhai (2018). In addition, based on a survey conducted at year 2013, Xu *et al*. concluded that surface Ω_A_ in BoHai and Yellow seas ranged from 2.0 to 3.8^42^, which compares well with our sea surface mean Ω_A_ of 3.3 in the East China.

### Future projections of ocean acidification in the East China

To have a better understanding of the ocean acidification conditions in the East China under different atmospheric CO_2_ scenarios, we performed four simulations under different RCP scenarios (Fig. 1a). With the continuous increasement in atmospheric CO_2_ content, the ocean keeps absorbing atmospheric CO_2_, leading to continuing acidification in the global ocean (see Supplementary Figs. S5 and S6). As shown in Figs. S5 and S6, in terms of $[{{\rm{C}}{\rm{O}}}_{3}^{2-}]$ and Ω_A_, ocean surface in the East China would experience greater ocean acidification than the tropical and high latitude ocean surface. The Arctic Ocean experiences the greatest reductions in sea surface pH, mainly due to the effects of rising temperature (Fig. 4b).

Simulated results show that responses of ocean acidification could be sensitive to the atmospheric CO_2_ scenarios used. For example, in the simulation under RCP2.6 scenario, by year 2300, the cumulative CO_2_ uptake for ocean in the East China is 19.7 PgC, leading to reductions in sea surface pH and $[{{\rm{C}}{\rm{O}}}_{3}^{2-}]$ by 1% and 13% (Fig. 1, Supplementary Table S1). In contrast, in the simulation under RCP8.5 scenario, by year 2300, the cumulative oceanic CO_2_ uptake is 23.4 PgC, resulting in reductions in surface pH and $[{{\rm{C}}{\rm{O}}}_{3}^{2-}]$ by 9% and 72%, respectively (Fig. 1, Supplementary Table S1).

**Figure 6:**
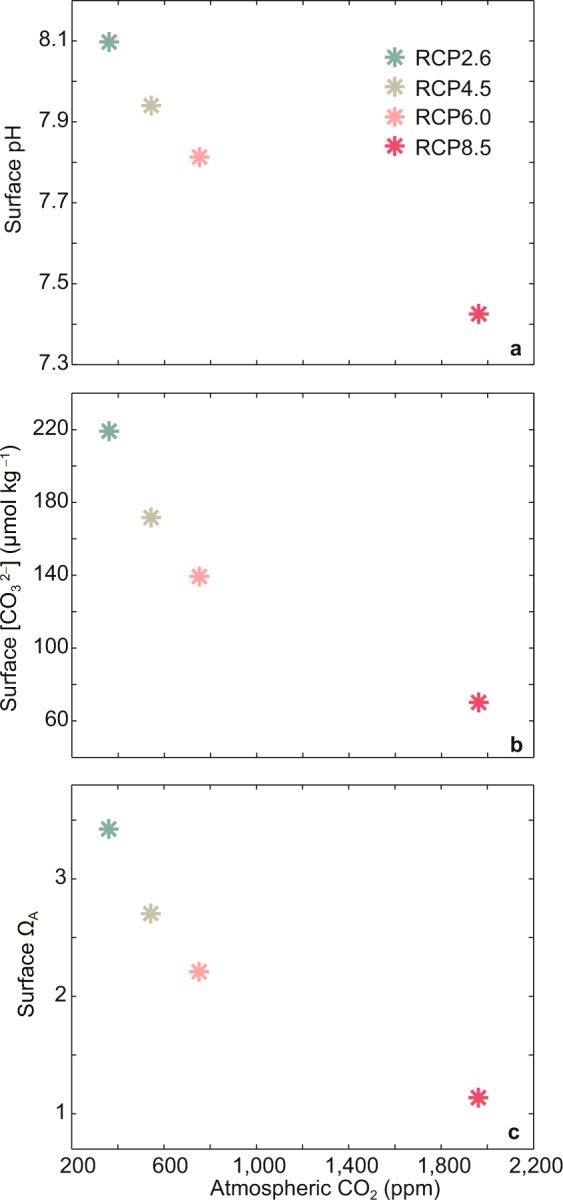
Prescribed atmospheric CO_2_ concentration against model-simulated ocean surface (**a**) pH, (**b**) $[{{\rm{C}}{\rm{O}}}_{3}^{2-}]$, and (**c**) Ω_A_ in the East China at year 2300. Results are shown for the four simulations using the four RCP scenarios depicted in Methods section, revealing the nonlinearity relationship between atmospheric CO_2_ scenario used and ocean acidification.

In addition, the relationship between atmospheric CO_2_ scenario used and ocean acidification in the East China is nonlinear (Fig. 6). For instance, at year 2300, for sea surface pH in the East China, ΔpH_RCP8.5-RCP6.0_/ΔCO_2 RCP8.5-RCP6.0_ = −3.2 × 10^−3^, ΔpH_RCP6.0-RCP4.5_/ΔCO_2 RCP6.0-RCP4.5_ = −6.1 × 10^−3^, while ΔpH_RCP4.5-RCP2.6_/ΔCO_2 RCP4.5-RCP2.6_ = −8.6 × 10^−3^ (Fig. 6, Table S1), indicating faster acidification rates under scenarios of lower atmospheric CO_2_ content. At year 2300, for sea surface $[{\rm{C}}{{\rm{O}}}_{3}^{2-}]$ in the East China, ${\Delta [{\rm{CO}}}_{3}^{2-}{]}_{{\rm{RCP8}}.5-{\rm{RCP6}}.0}$/ΔCO_2 RCP8.5-RCP6.0_ = −0.06, $\Delta {{[{\rm{CO}}}_{3}^{2-}]}_{{\rm{RCP6}}.0-{\rm{RCP4}}.5}$/ΔCO_2 RCP6.0-RCP4.5_ = −0.15, while ${\Delta [{\rm{CO}}}_{3}^{2-}{]}_{{\rm{RCP4}}.5-{\rm{RCP2}}.6}$/ΔCO_2 RCP4.5-RCP2.6_ = −0.26 (Fig. 6, Table S1), showing greater nonlinearity than surface pH. The nonlinearity between atmospheric CO_2_ scenario used and ocean acidification is noteworthy, which hints that if we aim to mitigate ocean acidification in the East China under a scenario of high atmospheric CO_2_ content, a deeper reduction of anthropogenic CO_2_ emission may be needed.

With the decrease of seawater $[{{\rm{C}}{\rm{O}}}_{3}^{2-}]$ in the East China, the aragonite saturation state (Ω_A_) would also diminish (Fig. 1g,h). We take the simulations under RCP8.5 and RCP4.5 scenarios for example, to investigate the possible effects of ocean acidification on seawater chemistry in the East China under intensive and medium atmospheric CO_2_ scenarios.

Under RCP8.5, due to the oceanic CO_2_ uptake and the resultant reduction in $[{{\rm{C}}{\rm{O}}}_{3}^{2-}]$, ocean Ω_A_ drops from the preindustrial value of 1.6 to 0.7 by year 2300, and seawater Ω_A_ would become less than 1 at nearly all ocean depths (Fig. 1h, Supplementary Fig. S7a and Table S1). The decreases in seawater Ω_A_ would pose threats to calcifying organisms over ocean in the East China. For example, seawater that surrounding coral reefs becomes more and more acidic, from surface to depth (see Supplementary Figs. S7a, and S8a–c). Aragonite is the main constituents of calcareous endoskeleton of corals, therefore, the corals surrounded by undersaturated seawater with respect to aragonite (Ω_A_ < 1) would encounter adverse impacts. There are also intriguing evidences that even in supersaturated seawater, CaCO_3_ also dissolves^3,51^. Seawater chemistry fields at different depths have different responses to oceanic CO_2_ uptake. For ocean in the East China, by year 2100, the reduction in Ω_A_ is 2.0 at depth of 17.5 m, which becomes 0.3 at depth of 642.5 m (see Supplementary Fig. S7a). Changes in ocean chemistry at depths lag behind changes in the surface ocean because of the long time scale associated with the penetration of CO_2_ into the deep ocean.

In comparison, in the simulation under RCP4.5 scenario, ocean Ω_A_ drops from the preindustrial value of 1.6 to 1.2 by year 2300 (Fig. 1h, Supplementary Table S1). Compared with simulated results under RCP8.5, simulation under RCP4.5 presents higher ocean Ω_A_ at years 2100 and 2300 (see Supplementary Figs. S7 and S8). In addition, from year 2100 to 2300, in simulation under RCP8.5, Ω_A_ at different depths continues to decrease, while in simulation under RCP4.5, the reductions in Ω_A_ at different depths, especially the decreases in surface Ω_A_ are slight (Fig. 1, Supplementary Figs. S7 and S8). Therefore, responses of ocean acidification in the East China would be sensitive to the changes in atmospheric CO_2_, demonstrating the important impacts of atmospheric CO_2_ changes on marine chemistry.

### Impacts of CO_2_-induced warming on ocean acidification in the East China

The impact of CO_2_-induced warming on ocean acidification in the East China could be inspected by comparing modeled ocean chemistry fields from simulations with and without CO_2_-induced warming (see Supplementary Table S2 and Fig. S9). Generally, CO_2_-induced warming would decrease the amount of ocean uptake of atmospheric CO_2_, mitigating ocean acidification (see Supplementary Table S2). Previous studies concluded that, this warming-induced reduction in oceanic CO_2_ uptake is mainly as a result of warming-induced decreases in CO_2_ solubility and ocean ventilation (ocean mixing and circulation)^9,52,53^. Effects of warming-induced changes in ocean biology processes (including phytoplankton growth and mortality rates, and detritus remineralization) offset with each other, therefore the total warming-induced biological impact on oceanic CO_2_ uptake and ocean acidification is small (refer to Cao and Zhang, 2017)^9^.

**Figure 7:**
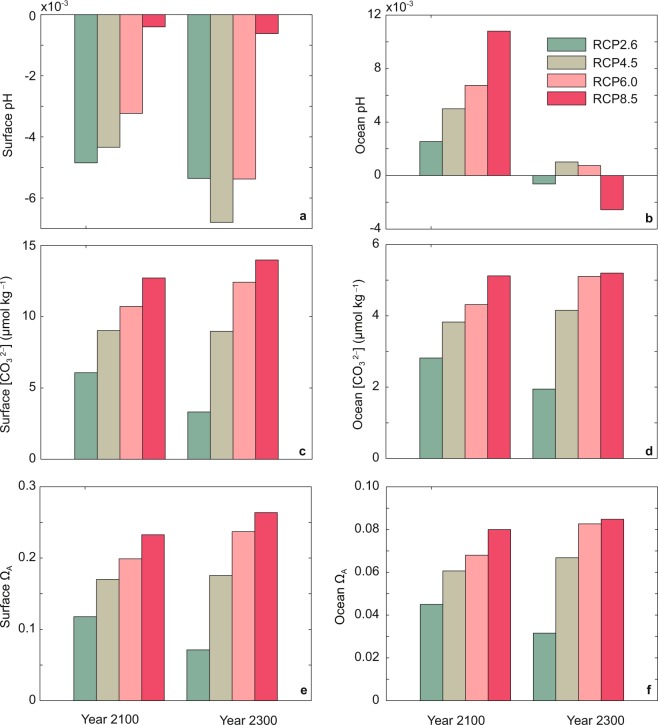
Model-simulated effects of CO_2_-induced warming at years 2100 and 2300 on (**a**) ocean surface pH, (**b**) ocean mean pH, (**c**) ocean surface $[{{\rm{C}}{\rm{O}}}_{3}^{2-}]$, (**d**) ocean mean $[{{\rm{C}}{\rm{O}}}_{3}^{2-}]$, (**e**) ocean surface Ω_A_, (**f**) ocean mean Ω_A_ in the East China. The effect of CO_2_-induced warming is represented by the difference between the results from simulations with and without warming effect. Results are shown for the four simulations using the four RCP scenarios depicted in Methods section.

For regional ocean in the East China, the responses of seawater pH and $[{{\rm{C}}{\rm{O}}}_{3}^{2-}]$ to CO_2_-induced warming are different (Fig. 7). The decoupled effects of CO_2_-induced warming on pH and $[{{\rm{C}}{\rm{O}}}_{3}^{2-}]$ are mainly due to their different thermodynamic dependence on temperature^39^ (Fig. 4). For pH, the direct effect of rising temperature tends to reduce seawater pH (Fig. 4b), while warming-induced reductions in CO_2_ solubility and ocean ventilation would suppress oceanic CO_2_ uptake and mitigate the decrease in ocean pH. Total influence of CO_2_-induced warming on seawater pH mainly depends on the relative importance of these two effects, i.e., thermodynamic effects of temperature increasing and warming-induced reductions in CO_2_ solubility and ocean ventilation. For ocean in the East China, relative to ocean depths, changes of pH in the ocean surface depend more on thermodynamic effects of rising temperature, resulting in decreased surface pH due to CO_2_-induced warming (Fig. 7a). At ocean depths, at year 2100, pH changes depend more on warming-induced reductions in CO_2_ solubility and ocean ventilation, leading to increased ocean pH due to CO_2_-induced warming (Fig. 7b). For instance, at year 2100, in simulation RCP4.5, for ocean in the East China, surface pH decreases by about 0.005 due to CO_2_-induced warming, whereas ocean mean pH increases by 0.005 due to CO_2_-induced warming (Fig. 7 and Table S2). For $[{{\rm{C}}{\rm{O}}}_{3}^{2-}]$ and Ω_A_, thermodynamic effects of temperature increasing and warming-induced reductions in CO_2_ solubility and ocean ventilation both act to mitigate the reductions in $[{{\rm{C}}{\rm{O}}}_{3}^{2-}]$ and Ω_A_ (Fig. 7c–f).

Compared to the individual effect of oceanic CO_2_ uptake, the effect of CO_2_-induced warming on ocean acidification in the East China is relatively small (see Supplementary Fig. S9). For instance, at year 2300, under RCP8.5, for ocean in the East China, CO_2_-induced surface $[{{\rm{C}}{\rm{O}}}_{3}^{2-}]$ decrease is 196 μmol kg^−1^ (${\Delta [{\rm{CO}}}_{3}^{2-}{]}_{2300-1800}$ under RCP8.5 without warming effect), whereas warming-induced surface $[{{\rm{C}}{\rm{O}}}_{3}^{2-}]$ increase is only 14 μmol kg^−1^ (Fig. 7, and Supplementary Table S2, Fig. S9).

## Discussion

In this study, we conduct model simulations to assess ocean acidification in the East China on the timescale of centuries. During preindustrial time-year 2017, under the atmospheric CO_2_ scenario that is based on observations, as atmospheric CO_2_ concentration increases, the global ocean experiences acidification, and the ocean surface in the East China is one of the most vulnerable areas to ocean acidification. By year 2017, sea surface pH in the East China drops from the preindustrial level of 8.20 to 8.06, corresponding to a 35% rise in [H^+^]. In addition, the decrease rate of surface pH becomes faster in the last two decades. The changes in surface seawater acidity largely result from CO_2_-induced changes in surface DIC, ALK, salinity and temperature, among which DIC plays the most important role (accounting for more than 82% reductions in surface pH, $[{{\rm{C}}{\rm{O}}}_{3}^{2-}]$ and Ω_A_). In future projections, responses of ocean acidification could be sensitive to the atmospheric CO_2_ scenarios chosen. By year 2300, simulated reductions in sea surface $[{{\rm{C}}{\rm{O}}}_{3}^{2-}]$ are 13% under RCP2.6, 32% under RCP4.5, 45% under RCP6.0, contrasted to 72% under RCP8.5.

Moreover, our simulated results show that the relationship between atmospheric CO_2_ scenario used and ocean acidification is nonlinear. This is important because if we want to mitigate ocean acidification in the East China under a scenario of high CO_2_ concentration, a deeper reduction of anthropogenic CO_2_ emission may be needed. Furthermore, our simulations show that with the continuous oceanic CO_2_ uptake, changes in deep ocean chemistry exhibits time lag relative to the surface ocean, due to the long time scale associated with the slow penetration of excess CO_2_ to the deep ocean. The long time scale for changes in deep ocean chemistry also indicates the urgent of deep reductions in anthropogenic CO_2_ emissions, to avoid continuous accumulation of CO_2_ at ocean depths. In addition, CO_2_-induced warming acts to mitigate the reductions in seawater $[{{\rm{C}}{\rm{O}}}_{3}^{2-}]$ and Ω_A_ in the East China, and the individual effect of oceanic CO_2_ uptake is much greater than the effect of CO_2_-induced warming on ocean acidification.

Under RCP8.5, for ocean in the East China, by year 2300, with the decrease of ocean $[{{\rm{C}}{\rm{O}}}_{3}^{2-}]$, aragonite saturation state (Ω_A_) would drop from its initial value of 1.6 to 0.7, and seawater Ω_A_ would become undersaturated (Ω_A_ < 1) at nearly all ocean depths. Ocean in the East China is the habitat of numerous amounts of corals, fish, shellfish, and other calcifying organisms, such as crustaceans (e.g., penaeus, scylla serrata), gastropods (e.g., cypraea tigris, cassis cornuta), coccolithophorids (e.g., chrysophyta), including rare species. These calcifying organisms may not be able to acclimate to the reduction in seawater Ω_A_. By carrying out short-term pCO_2_/pH perturbation experiments, Wu and Gao concluded that, the combined impacts of seawater acidification and solar UV changes could also inhibit photosynthesis in the China seas^54^. Morphology, physiology and behavior of some other marine organisms (e.g., molluscs, cnidarians) could also be impacted by ocean acidification^55^. Therefore, ocean acidification in the East China could have adverse effects on fundamental biochemical processes and marine ecosystems. Since ocean in the East China plays an important role in global fishery and marine aquaculture industries, its trend in ocean acidification would have far-reaching consequences for the millions of people that depend on the food and other resources in the ocean for their livelihoods^56^.

Our study has investigated the CO_2_-induced ocean acidification conditions in the East China on timescales of centuries by using an Earth system model. Some processes or feedbacks that are not considered in this study may also have effects on ocean acidification^16,17,57^. For example, ocean acidification tends to suppress the calcification rate of some marine calcifying organisms, increasing surface ocean alkalinity and reducing seawater acidity^16^. This study also does not include the interactive feedbacks between ocean acidification and CaCO_3_ in the sediments, which is considered to reduce the chemistry change extent in the deep ocean on timescales longer than a millennium^58–60^.

In this study, based on model-simulated results, we diagnose ocean acidification in the East China during preindustrial time-year 2017, and highlight the potential future ocean acidification condition over timescales of centuries. Meanwhile, this study tries to provide useful information about the changes in future marine biogeochemical environment. Further observational and modeling studies would be required to develop a better understanding of the ocean carbon cycle and marine biogeochemistry, which is crucial for more reliable projections of future ocean acidification and its impacts on marine ecosystems.

## Methods

### Model description

In this study, we utilize the University of Victoria Earth System Climate Model (UVic ESCM) version 2.9, an intermediate complexity Earth system model^46^. The UVic model consists of an energy-moisture balance atmospheric model^61^, a 3D ocean general circulation model^62^, a thermodynamic/dynamic sea ice model^63,64^, and ocean and land carbon cycle models^46,65–67^. The horizontal resolution of the UVic model is 1.8° (latitude) × 3.6° (longitude), which is similar to the resolutions of most coupled Atmosphere-Ocean General Circulation Models (AOGCMs)^45^. The ocean model of UVic is the Modular Ocean Model (MOM) version 2.2 with 19 vertical levels, developed by the Geophysical Fluid Dynamics Laboratory^62^.

The ocean carbon cycle model of UVic comprises inorganic and organic carbon cycle modules. The inorganic carbon cycle is based on the Ocean Carbon-Cycle Model Intercomparison Project (OCMIP)^68^. The organic carbon cycle is represented by a nutrient-phytoplankton-zooplankton-detritus (NPZD) ocean ecosystem/biogeochemical model^46,67^. The land surface model and vegetation model are represented by Met Office Surface Exchange Scheme (MOSES) and Top-down Representation of Interactive Foliage and Flora Including Dynamics (TRIFFID) vegetation model, developed by the Hadley Center^65,66^.

The UVic model has participated in a series of international model intercomparison projects, including an intercomparison of Earth System Models of Intermediate Complexity (EMICs) undertaken in support of the Intergovernmental Panel on Climate Change Fifth Assessment Report (IPCC AR5)^69,70^, the Coupled Carbon Cycle Climate Model Intercomparison Project (CMIP)^71^, the Paleoclimate Modeling Intercomparison Project (PMIP)^72^, and a few thermohaline circulation experiments^73,74^. The UVic model has been widely used in the studies concerning future evolutions of the ocean biogeochemical cycles^46^, interactions between global carbon cycle and climate change^71^, and projections of ocean acidification^75,76^.

The UVic model also has been widely used to investigate spatial distributions of physical and biogeochemical fields in both paleo and contemporary climate studies. For instance, Alexander *et al*. (2015) conducted model simulations with the UVic model and suggested that the spatial changes in carbonate dissolution during the Palaeocene-Eocene Thermal Maximum (PETM) could be explained by corrosive deep water spreading from the North Atlantic Ocean^77^. Bralower *et al*. (2014) found that UVic-simulated temperature, salinity, calcite saturation state, and dissolved O_2_ and PO_4_ at different ocean depths are generally agree with integrated published data from the onset of the PETM at a coastal Site 690, Maud Rise in the Southern Ocean^78^. Xiao *et al*. (2012) used the UVic model to examine contributions of different climate forcings to surface air temperature over East China in the past millennium, and found the model successfully reproduced the observational-based temperature variation of East China^79^. Xiao *et al*. (2012) also suggested that the mean error of air temperature simulated by the UVic model could be even smaller than that by many famous complex models^45,79^. In addition, Meissner^80^, Weaver *et al*.^81^, Spence and Weaver^82^, and Muglia and Schmittner^83^ conducted UVic simulations to investigate variations in Atlantic meridional overturning circulation. Therefore, it is feasible to use the UVic model to quantify ocean acidification in the East China induced by oceanic uptake of anthropogenic CO_2_ on timescales of centuries.

### Simulation experiments

The UVic model was first spun up for 10,000 model years with a fixed preindustrial CO_2_ concentration of 280 ppm to reach a quasi-equilibrium state of carbon cycle and climate system. Then, using this preindustrial state as an initial condition for the calendar year of 1800, two sets of four 500-year transient simulations are performed (i.e., from year 1800 to 2300). In the first set of simulations, rising atmospheric CO_2_ concentration affects both the ocean carbon cycle and atmospheric radiation. While in the second set of simulations, rising atmospheric CO_2_ concentration is not allowed to affect atmospheric radiation, that is, the ocean carbon cycle would not be impacted by CO_2_-induced warming. Each set of experiments include four simulations. From year 1800 to 2017, atmospheric CO_2_ concentration data are taken from observational-based estimates, and after 2017, CO_2_ concentrations are taken from the Representation Concentration Pathway scenarios (RCPs) and their extensions up to year 2300^84^ (Fig. 1a). The four scenarios used are RCP2.6, RCP4.5, RCP6.0, and RCP8.5, based on different mitigation policies for greenhouse gases^84,85^. The numbers after “RCP” represent that by year 2100, the radiative forcing reaches 2.6, 4.5, 6.0, or 8.5 W m^−2^, respectively. Refer to Meinshausen *et al*. (2011) for detailed descriptions of these RCP scenarios^84^.

### Analysis of ocean chemistry fields

In this study, we calculate ocean carbonate chemistry fields, including seawater pH, $[{\rm{C}}{{\rm{O}}}_{3}^{2-}]$ and Ω_A_, based on equations from the OCMIP-3 project (http://ocmip5.ipsl.jussieu.fr/OCMIP/). We use UVic-simulated ocean temperature, salinity, DIC, ALK, and observational-based estimates of ocean phosphate and silicate concentrations from the Global Ocean Data Analysis Project (GLODAP)^50^. Here comes the calculations^86^.

Thermodynamic carbonate chemistry system can be generally represented by the following 6 variables: [H^+^], DIC, ALK, [CO_2_], $[{{\rm{HCO}}}_{3}^{-}]$, and $[{\rm{C}}{{\rm{O}}}_{3}^{2-}]$. Here, [CO_2_] is the sum of concentrations of CO_2_ (aq) (aqueous carbon dioxide) and H_2_CO_3_ (carbonate acid), and $[{{\rm{HCO}}}_{3}^{-}]$ represents bicarbonate ion concentration. Additionally, equilibrium expressions for H_2_CO_3_ dissociation are given by

3 $${{\rm{K}}}_{1}^{\ast }=\frac{{[{\rm{HCO}}}_{3}^{-}{][{\rm{H}}}^{+}]}{{[{\rm{CO}}}_{2}]}$$

4 $${{\rm{K}}}_{2}^{\ast }=\frac{{[{\rm{CO}}}_{3}^{2-}{][{\rm{H}}}^{+}]}{{[{\rm{HCO}}}_{3}^{-}]}$$

Expressions for DIC and ALK are given by

5 $${\rm{DIC}}={[{\rm{CO}}}_{2}]+{[{\rm{HCO}}}_{3}^{-}]+{[{\rm{CO}}}_{3}^{2-}]$$

6 $${\rm{ALK}}\approx {[{\rm{HCO}}}_{3}^{-}]+2{[{\rm{CO}}}_{3}^{2-}]+{[{\rm{OH}}}^{-}]-{[{\rm{H}}}^{+}]$$

Based on the above 6 variables ([H^+^], DIC, ALK, [CO_2_], ${[{\rm{HCO}}}_{3}^{-}]$, and $[{{\rm{C}}{\rm{O}}}_{3}^{2-}]$), and 4 equations (Eqs. (3–6)) of ocean carbonate chemistry, given 2 known variables, we can calculate the rest 4 variables^87^. Therefore, changes in temperature, salinity (temperature and salinity changes would affect ${{\rm{K}}}_{1}^{\ast }$ and ${{\rm{K}}}_{2}^{\ast }$), DIC, and ALK, would result in changes in pH (pH = −log_10_[H^+^]) and $[{{\rm{C}}{\rm{O}}}_{3}^{2-}]$, i.e., changes in seawater acidity.

## Supplementary information

Supplementary Information

## Data Availability

All data generated or analysed during this study are included in this published article (and its Supplementary Information files).
