# Supplementary material for: Simulated CO2-induced ocean acidification for ocean in the East China: historical conditions since preindustrial time and future scenarios
Source: Sci Rep. 2019 Dec 6;9:18559. doi: 10.1038/s41598-019-54861-0 (PMC6897940; doi:10.1038/s41598-019-54861-0)
Supplement: Supplementary file 1 — Supplementary Information [file 41598_2019_54861_MOESM1_ESM.pdf]

Simulated CO_2-induced ocean acidification for ocean in the East China: historical conditions since preindustrial time and future scenarios

Han Zhang[1,2] and Kuo Wang[1,*]

1 Zhejiang Climate Center, Hangzhou, Zhejiang 310017, China

2 Department of Atmospheric Sciences, School of Earth Sciences, Zhejiang University, Hangzhou, Zhejiang 310027, China

[*] Corresponding author (E-mail: wangkuo.climate@qq.com)

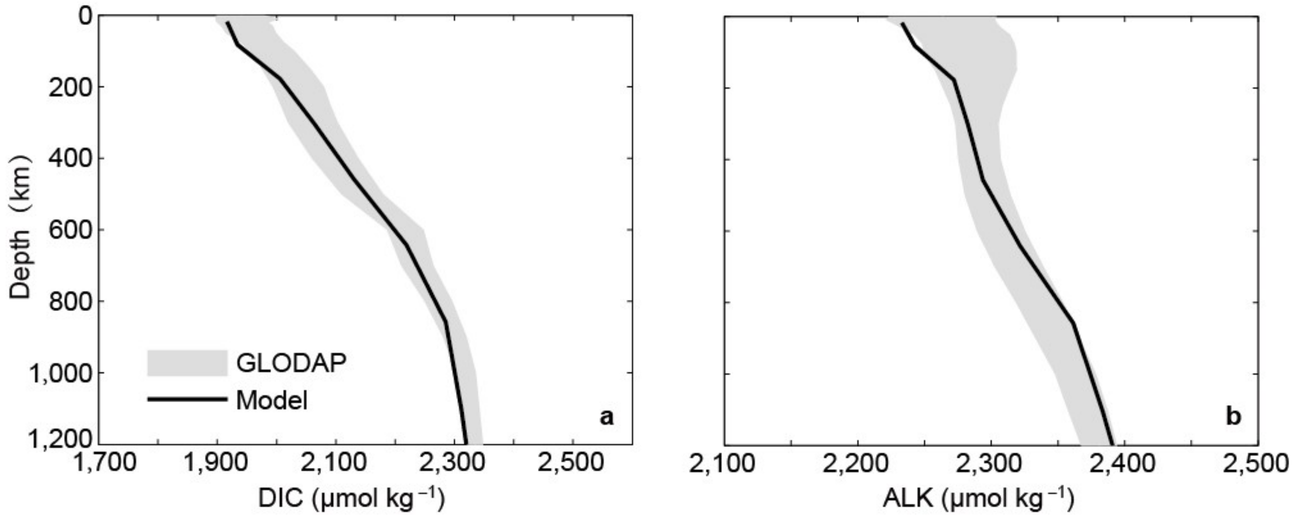

Supplementary Figure S1. Modeled mean vertical profiles for ocean in the East China of (a) dissolved inorganic carbon (DIC) and (b) alkalinity (ALK) (1990-1999 average) compared with observational-based GLODAP data (gray shaded areas represent estimated uncertainties)[1].

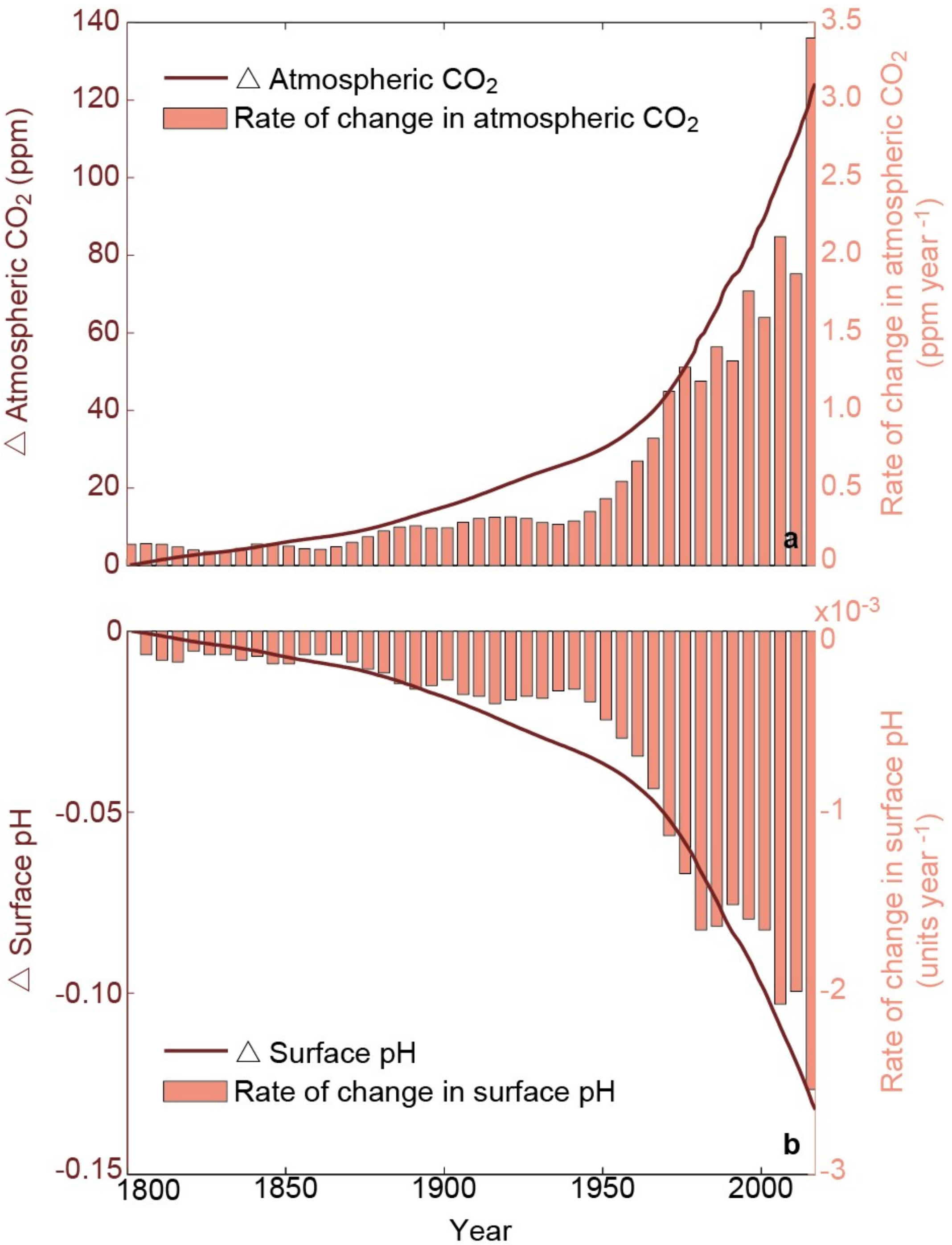

Supplementary Figure S2. Time series of simulated annual mean changes in (a) atmospheric CO_2 concentration, and (b) ocean surface pH in the East China from 1800 to 2017. Brown lines represent the change relative to year 1800, while pink bars represent the rate of change (the change relative to the last year). As shown in the figure, the reduction rate of surface pH agrees well with the increasing rate of atmospheric CO_2 concentration.

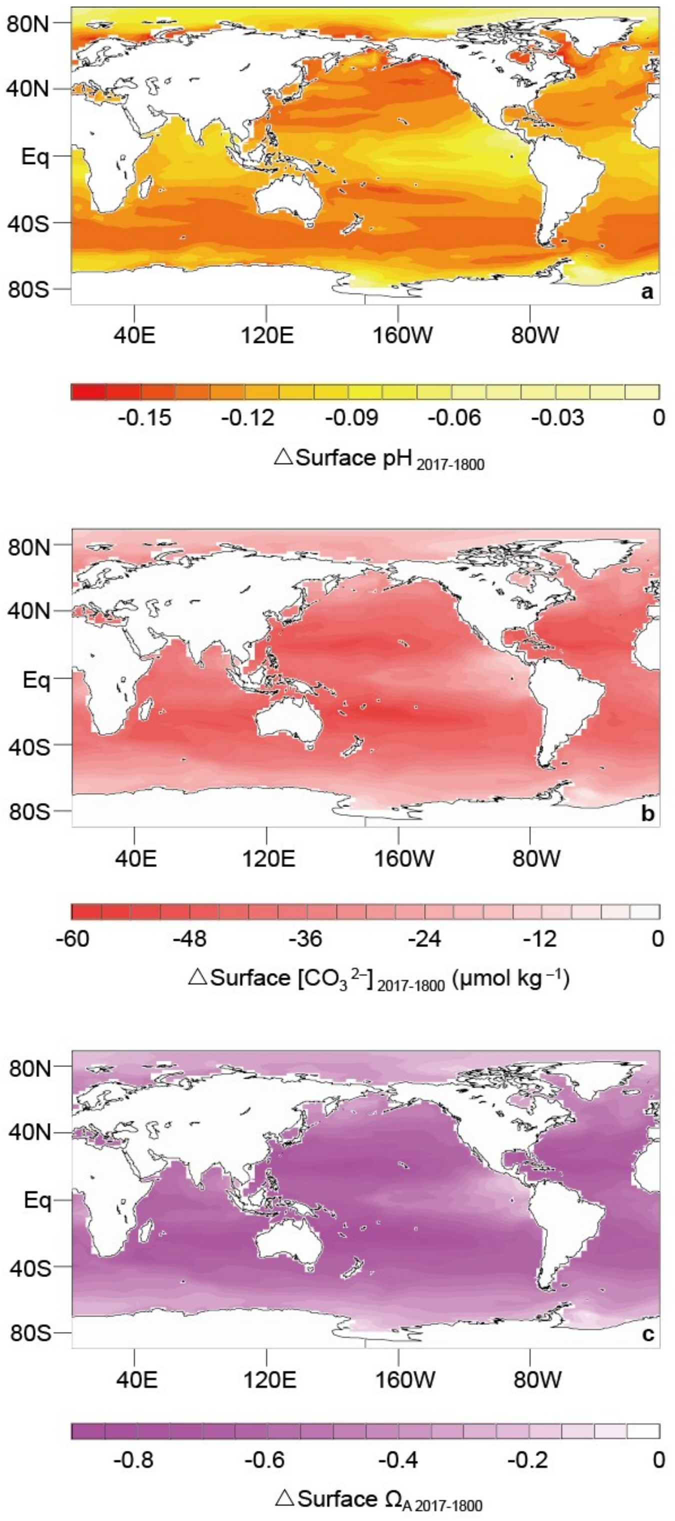

Supplementary Figure S3. Simulated changes at year 2017 in the spatial distributions of ocean surface (a) pH, (b) $[CO_3^{2-}]$, and (c) Ω_A for the global ocean (relative to 1800). The figures are generated using UV-CDAT version 2.5.0 (http://uvcdat.llnl.gov/).

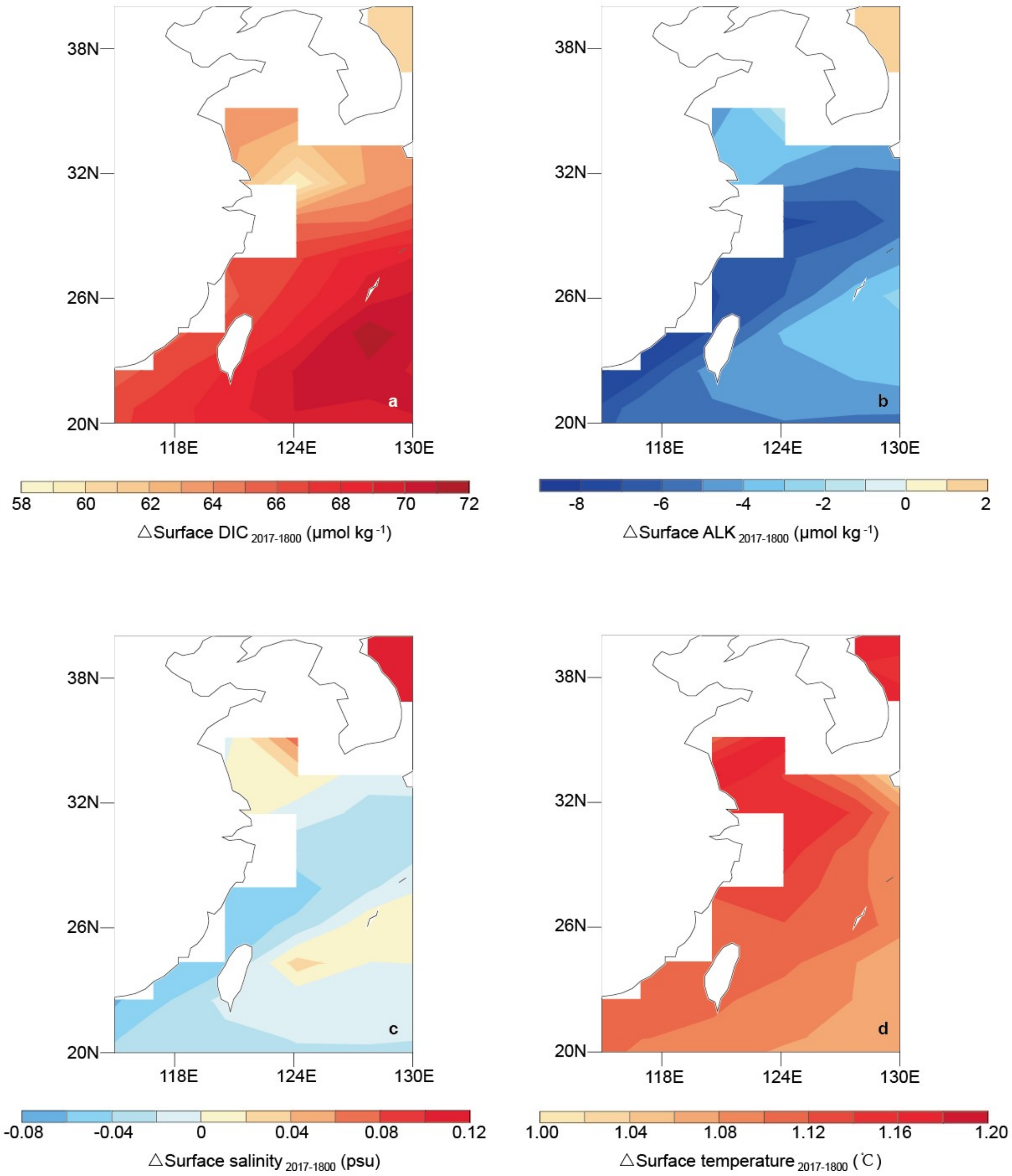

Supplementary Figure S4. Spatial distributions of simulated changes (relative to 1800) at year 2017 in ocean surface (a) DIC, (b) ALK, (c) salinity, and (d) temperature over ocean in the East China. The figures are generated using UV-CDAT version 2.5.0 (http://uvcdat.llnl.gov/).

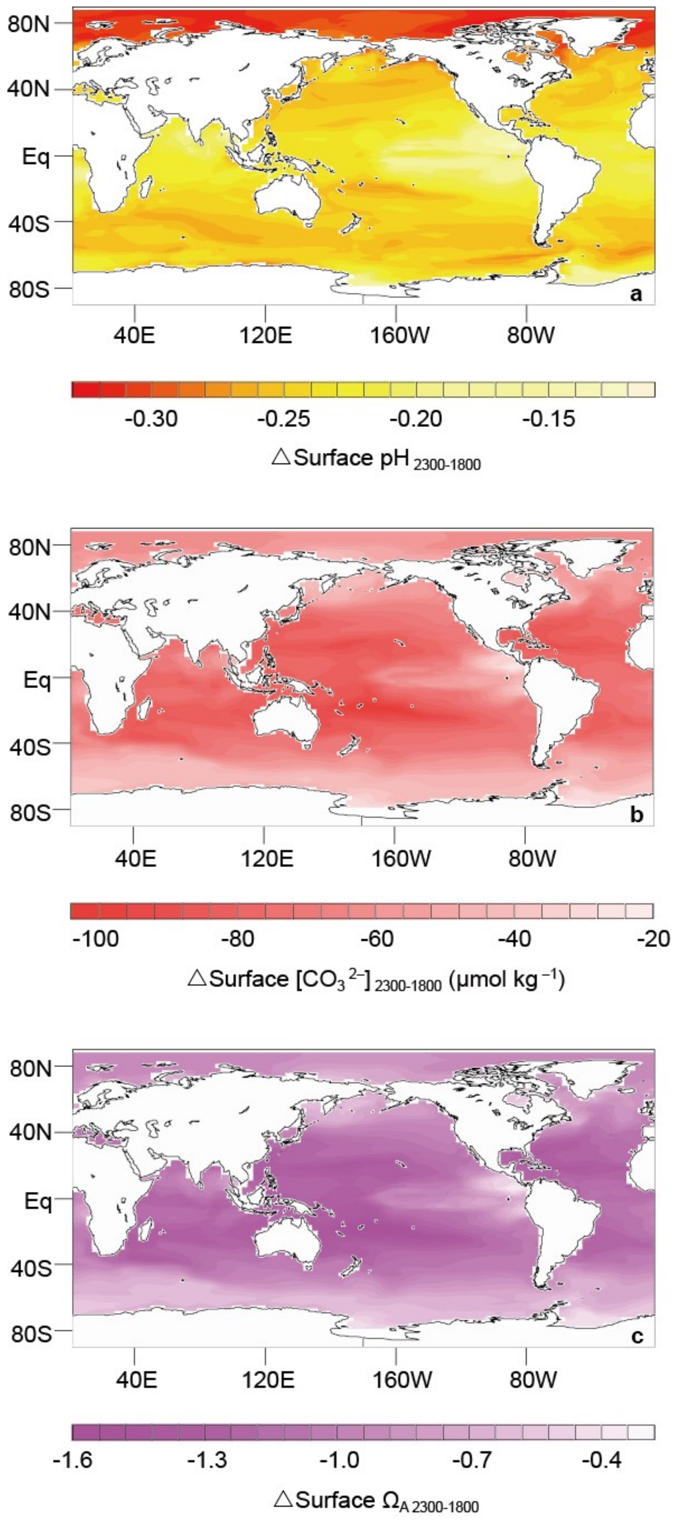

Supplementary Figure S5. Simulated changes at year 2300 in the spatial distributions of ocean surface (a) pH, (b) $[CO_3^{2-}]$, and (c) Ω_A for the global ocean (relative to 1800) under RCP4.5. The figures are generated using UV-CDAT version 2.5.0 (http://uvcdat.llnl.gov/).

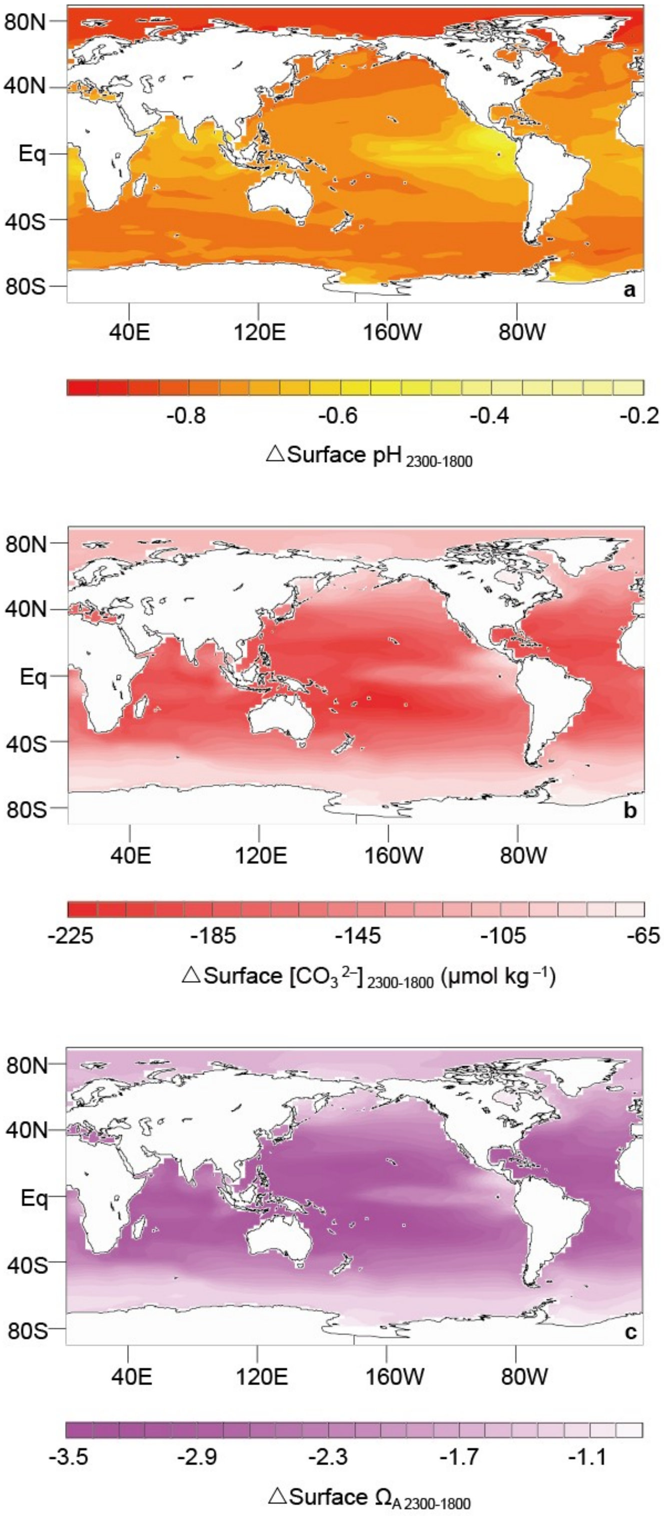

Supplementary Figure S6. Simulated changes at year 2300 in the spatial distributions of ocean surface (a) pH, (b) $[CO_3^{2-}]$, and (c) Ω_A for the global ocean (relative to 1800) under RCP8.5. The figures are generated using UV-CDAT version 2.5.0 (http://uvcdat.llnl.gov/).

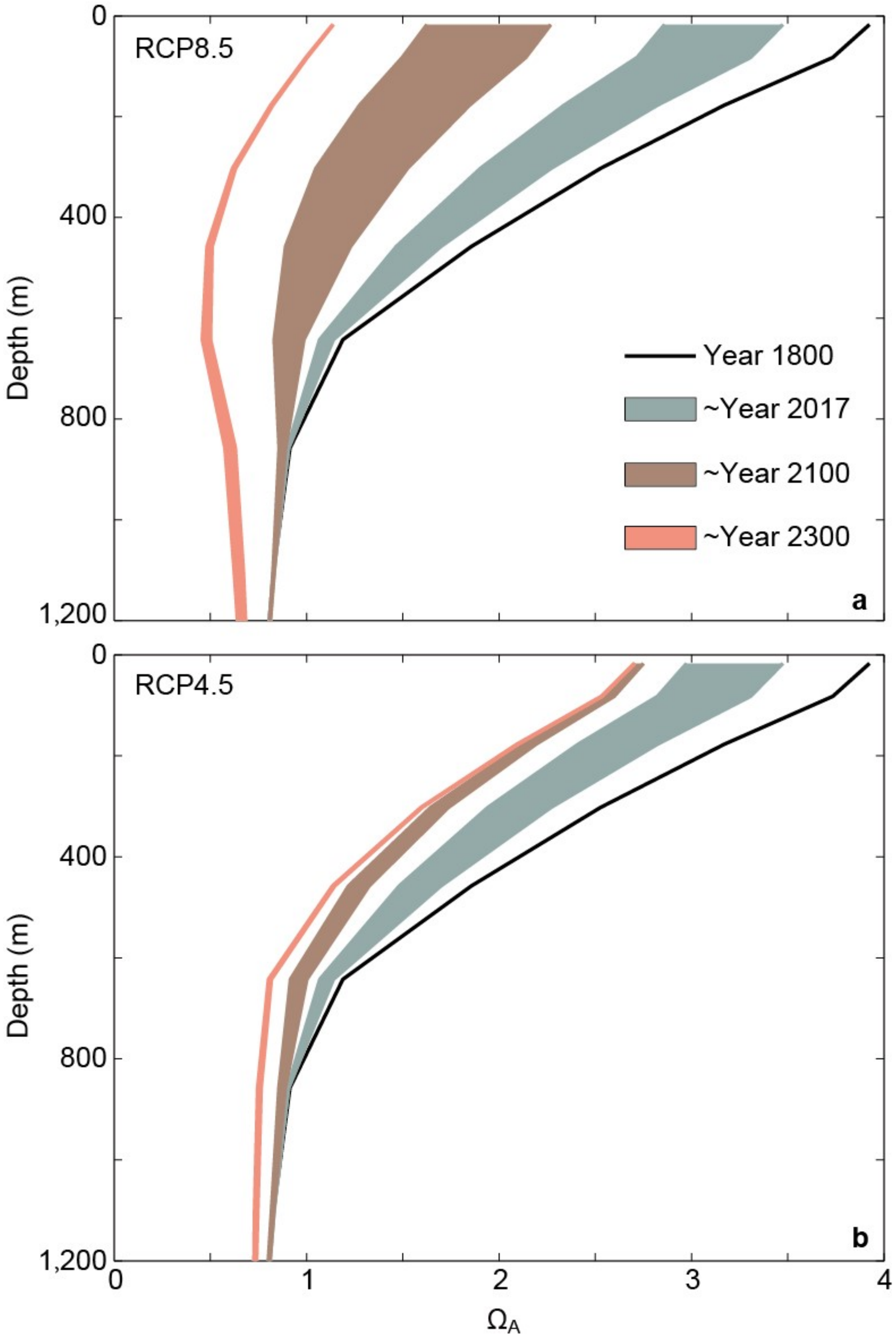

Supplementary Figure S7. Modeled mean vertical profiles of Ω_A for ocean in the East China at years 1800, ~2017 (1992-2042), ~2100 (2075-2125) and ~2300 (2275-2325) under (a) RCP8.5 and (b) RCP4.5.

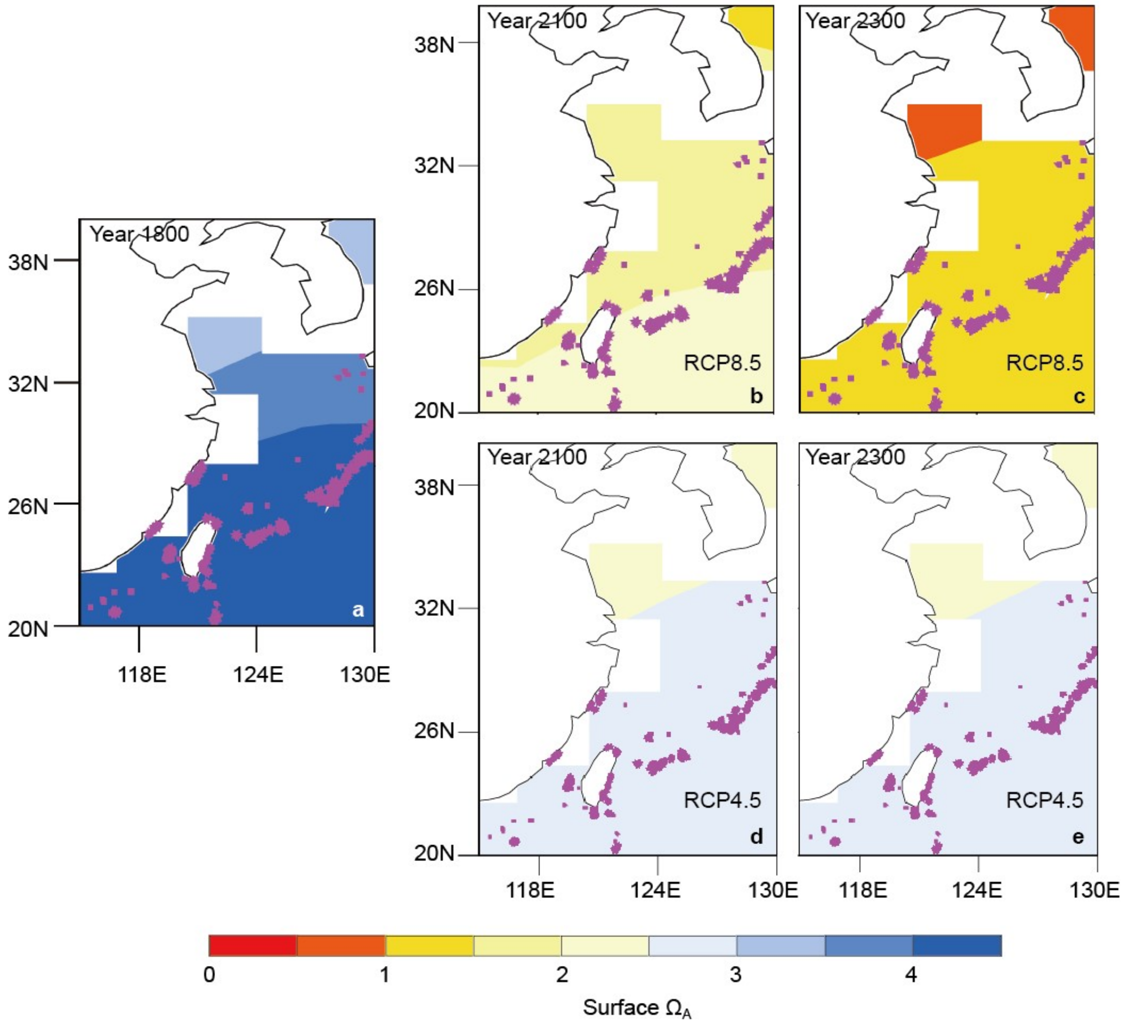

Supplementary Figure S8. Spatial distributions of surface Ω_A at years (a) 1800, (b, d) 2100, and (c, e) 2300 and locations of coral reefs over ocean in the East China. Results shown are from simulations (b, c) RCP8.5 and (d, e) RCP4.5. Purple points over plotted on the maps represent the locations of coral reefs. The longitude and latitude information are from Freiwald et al. (2017)[1], UNEP-WCMC (2018)[3], and ReefBase (2019)[4]. The figures are generated using UV-CDAT version 2.5.0 (http://uvcdat.llnl.gov/).

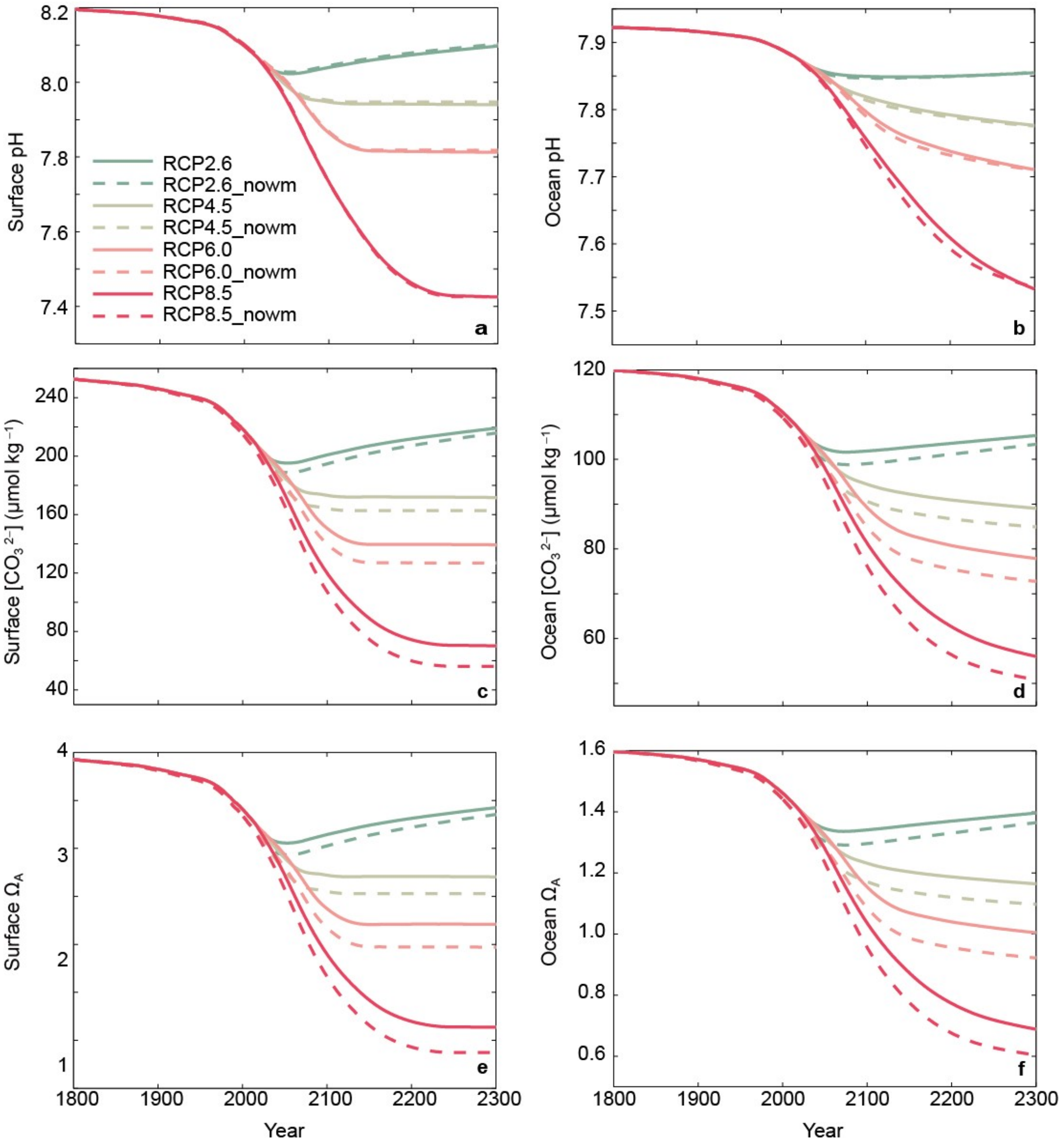

Supplementary Figure S9. Model-simulated time series of annual mean variable of (a) ocean surface pH, (b) ocean mean pH, (c) ocean surface $[CO_3^{2-}]$, (d) ocean mean $[CO_3^{2-}]$, (e) ocean surface Ω_A, (f) ocean mean Ω_A in the East China. Results are shown for the two sets of simulations using the four RCP scenarios, and the legend with or without the suffix "nowm" denotes the simulations with or without CO_2-induced warming (refer to Methods section for detailed descriptions).

Supplementary Table S1. Model-simulated key ocean chemistry fields change (relative to year 1800) at year 2017 (first numbers), 2100 (second numbers), and 2300 (third numbers) in the East China. Results are shown for the four simulations using the four RCP scenarios depicted in the Method section.

	**RCP2.6 2017/2100/2300**	**RCP4.5 2017/2100/2300**	**RCP6.0 2017/2100/2300**	**RCP8.5 2017/2100/2300**
**Cumulative ocean CO_2 uptake (PgC)**	8.7/12.2/19.7	8.7/12.6/20.7	8.7/12.8/21.6	8.7/13.3/23.4
**Atmospheric CO_2 (ppm)**	124/140/80	124/258/262	124/389/471	124/655/1681
**Sea surface pH**	-0.13/-0.16/-0.10	-0.13/-0.25/-0.26	-0.13/-0.33/-0.38	-0.13/-0.46/-0.77
**Ocean mean pH**	-0.04/-0.07/-0.07	-0.04/-0.10/-0.15	-0.04/-0.12/-0.21	-0.04/-0.16/-0.39
**Sea surface $[CO_3^{2-}]$ (μmol kg^{-1})**	-45/-52/-33	-45/-79/-81	-45/-102/-113	-45/-133/-182
**Ocean mean $[CO_3^{2-}]$ (μmol kg^{-1})**	-13/-18/-15	-13/-25/-31	-13/-31/-42	-13/-39/-64
**Sea surface Ω_A**	-0.7/-0.8/-0.5	-0.7/-1.2/-1.2	-0.7/-1.6/-1.7	-0.7/-2.0/-2.8
**Ocean mean Ω_A**	-0.2/-0.3/-0.2	-0.2/-0.4/-0.4	-0.2/-0.4/-0.6	-0.2/-0.6/-0.9

Supplementary Table S2. Model-simulated key ocean chemistry fields change (relative to year 1800) at year 2100 and 2300 for the simulations with (first numbers) and without (second numbers) CO_2-induced warming for ocean in the East China. Results are shown for the four simulations using the four RCP scenarios depicted in the Method section.

	**RCP2.6**		**RCP4.5**		**RCP6.0**		**RCP8.5**	
	**2100**	**2300**	**2100**	**2300**	**2100**	**2300**	**2100**	**2300**
**Cumulative ocean CO_2 uptake (PgC)**	12.2/ 12.7	19.7/ 20.9	12.6/ 13.2	20.7/ 22.4	12.8/ 13.4	21.6/ 23.2	13.3/ 13.9	23.4/ 24.7
**Sea surface pH**	-0.155/ -0.151	-0.098/ -0.093	-0.248/ -0.243	-0.255/ -0.248	-0.331/ -0.327	-0.382/ -0.377	-0.462/ -0.461	-0.770/ -0.769
**Ocean mean pH**	-0.073/ -0.075	-0.067/ -0.067	-0.103/ -0.108	-0.146/ -0.147	-0.125/ -0.131	-0.211/ -0.212	-0.165/ -0.176	-0.389/ -0.387
**Sea surface $[CO_3^{2-}]$ (μmol kg^{-1})**	-52/ -58	-33/ -37	-79/ -88	-81/ -90	-102/ -113	-113/ -126	-133/ -145	-182/ -196
**Ocean mean $[CO_3^{2-}]$ (μmol kg^{-1})**	-18/ -21	-15/ -16	-25/ -29	-31/ -35	-31/ -35	-42/ -47	-39/ -44	-64/ -69
**Sea surface Ω_A**	-0.8/ -0.9	-0.5/ -0.6	-1.2/ -1.4	-1.2/ -1.4	-1.6/ -1.8	-1.7/ -2.0	-2.0/ -2.3	-2.8/ -3.1
**Ocean mean Ω_A**	-0.26/ -0.30	-0.20/ -0.23	-0.37/ -0.43	-0.43/ -0.50	-0.44/ -0.51	-0.59/ -0.68	-0.56/ -0.64	-0.91/ -0.99

References

1. Key, R. M. *et al.* A global ocean carbon climatology: Results from Global Data Analysis Project (GLODAP). *GLOBAL BIOGEOCHEM CY*. **18** (4), GB4031 (2004).

2. Freiwald, A. *et al.*. Global distribution of cold-water coral reefs (version 5.0). Fifth update to the dataset in Freiwald et al. (2004) by UNEP-WCMC, in collaboration with Andre Freiwald and John Guinotte. Cambridge, UK: UN Environment World Conservation Monitoring Centre, [2019-2]. http://data.unep-wcmc.org/datasets/3 (2017).

3. UNEP-WCMC, WorldFish Centre, WRI, TNC. Global distribution of warm-water coral reefs, compiled from multiple sources including the Millennium Coral Reef Mapping Project. Version 4.0. Includes contributions from IMaRS-USF and IRD (2005), IMaRS-USF (2005) and Spalding et al. (2001). Cambridge, UK: UN Environment World Conservation Monitoring Centre, [2019-4]. http://data.unep-wcmc.org/datasets/1 (2018).

4. ReefBase. A global information system for coral reefs. [2019-4]. http://www.reefbase.org (2019).
